# Netrin-1 Alters Adipose Tissue Macrophage Fate and Function in Obesity

**DOI:** 10.20900/immunometab20190010

**Published:** 2019-08-07

**Authors:** Monika Sharma, Martin Schlegel, Emily J. Brown, Brian E. Sansbury, Ada Weinstock, Milessa S. Afonso, Emma M. Corr, Coen van Solingen, Lianne C. Shanley, Daniel Peled, Ravichandran Ramasamy, Ann Marie Schmidt, Matthew Spite, Edward A. Fisher, Kathryn J. Moore

**Affiliations:** 1 Department of Medicine, New York University School of Medicine, New York, NY 10016, USA; 2 Center for Experimental Therapeutics and Reperfusion Injury, Department of Anesthesiology, Perioperative and Pain Medicine, Brigham and Women’s Hospital and Harvard Medical School, Boston, MA 02115, USA; 3 Department of Cell Biology, New York University School of Medicine, New York, NY 10016, USA

**Keywords:** obesity, inflammation, macrophage, neuroimmune, guidance molecule, chemostasis, visceral adipose

## Abstract

Macrophages accumulate prominently in the visceral adipose tissue (VAT) of obese humans and high fat diet (HFD) fed mice, and this is linked to insulin resistance and type II diabetes. While the mechanisms regulating macrophage recruitment in obesity have been delineated, the signals directing macrophage persistence in VAT are poorly understood. We previously showed that the neuroimmune guidance cue netrin-1 is expressed in the VAT of obese mice and humans, where it promotes macrophage accumulation. To better understand the source of netrin-1 and its effects on adipose tissue macrophage (ATM) fate and function in obesity, we generated mice with myeloid-specific deletion of netrin-1 (*Ntn1*^fl/fl^ *LysMCre*^+/–^; Ntn1^Δmac^). Interestingly, Ntn1^Δmac^ mice showed a modest decrease in HFD-induced adiposity and adipocyte size, in the absence of changes in food intake or leptin, that was accompanied by an increase in markers of adipocyte beiging (*Prdm16*, UCP-1). Using single cell RNA-seq, combined with conventional histological and flow cytometry techniques, we show that myeloid-specific deletion of netrin-1 caused a 50% attrition of ATMs in HFD-fed mice, particularly of the resident macrophage subset, and altered the phenotype of residual ATMs to enhance lipid handling. Pseudotime analysis of single cell transcriptomes showed that in the absence of netrin-1, macrophages in the obese VAT underwent a phenotypic switch with the majority of ATMs activating a program of genes specialized in lipid handling, including fatty acid uptake and intracellular transport, lipid droplet formation and lipolysis, and regulation of lipid localization. Furthermore, Ntn1^Δmac^ macrophages had reduced expression of genes involved in arachidonic acid metabolism, and targeted LCMS/MS metabololipidomics analysis revealed decreases in proinflammatory eicosanoids (5-HETE, 6-*trans* LTB_4_, TXB_2_, PGD_2_) in the obese VAT. Collectively, our data show that targeted deletion of netrin-1 in macrophages reprograms the ATM phenotype in obesity, leading to reduced adipose inflammation, and improved lipid handling and metabolic function.

## INTRODUCTION

Obesity triggers a poorly understood systemic immune response characterized by the recruitment of macrophages and other immune cells to key metabolic tissues [1]. This low-grade inflammation is causally linked to insulin resistance and type 2 diabetes, and related health complications including hepatic and cardiovascular disease [1]. The worldwide prevalence of obesity has doubled since 1980, and there is an urgent need for new therapeutics for this growing pandemic [2]. The recognition that inflammation and metabolic dysfunction are so closely intertwined has prompted efforts directed towards developing immune-directed therapies for the treatment of obesity and its sequelae.

The origins of adipose tissue inflammation during obesity remain poorly understood, but are thought to include altered cellular lipid metabolism and extracellular lipid deposition, changes in intestinal permeability, and oxidative and mechanical stresses. The healthy adipose tissue is populated by both innate and adaptive immune cells, with macrophages accounting for approximately 10% of the cells in visceral adipose tissue depots of lean mice [3,4]. In obese mice and humans, macrophage numbers rise dramatically, reaching up to 50% of all cells in visceral adipose tissue [4]. This expansion of the macrophage population occurs through both recruitment of bone marrow-derived monocytes in response to adipose-derived chemotactic factors (e.g., CCL2 [5], semaphorin 3E [6]), as well as local proliferation of adipose tissue macrophages [7,8]. Studies in subjects with type 2 diabetes treated with the insulin-sensitizing thiazolidinediones have shown a correlation between improvements in systemic insulin resistance and reduction in adipose tissue macrophage content and inflammatory factors [9,10]. In addition, studies in mice showed that reducing macrophage accumulation in adipose and other metabolic tissues ameliorate diet-induced insulin resistance [11]. Targeting of factors that regulate monocyte recruitment to tissues (e.g., the chemokine CCL2) or macrophage inflammatory gene programs (e.g., NF-kB or JNK signaling component) have been shown to reduce visceral adipose tissue inflammation and improve insulin sensitivity in high fat diet (HFD) mice [12–14]. However, the dual requirement for such pathways in host pathogen defense and tissue repair may hinder their translation into therapeutics [15–17]. The development of novel therapies targeting chronic macrophage accumulation in metabolic tissues will thus require a better understanding of the mechanisms regulating tissue macrophage persistence.

Early studies suggested that the lean adipose tissue was populated with macrophages resembling alternatively activated M2-like macrophages [3,4], and that excess lipid and hypoxic conditions in the obese adipose tissue environment polarized recruited macrophages to an inflammatory state. Such inflammatory or M1-like macrophages were thought to elaborate proinflammatory mediators (e.g., tumor necrosis factor alpha; TNF-α [18], interleukin-1 beta; IL-1β [19,20], monocyte chemoattractant protein; MCP-1/CCL2 [12], and bioactive lipid mediators (e.g., prostaglandins and leukotrienes [21]) that potentiated local and systemic inflammation, and ultimately, insulin resistance. More recent studies using advanced macrophage tracing techniques and single cell RNA-sequencing indicate that not all macrophages in the obese adipose tissue are proinflammatory, with metabolic stress giving rise to non-classical activation states and those specialized to a high lipid environment [22–25]. Notably, adipose tissue macrophages have been shown to be important for local lipid homeostasis by mediating the uptake of excess fatty acid and triacylglyceride released from adipocytes via canonical lipolysis and exosomal secretion, respectively [22,26–28]. Although adipose tissue macrophages are now recognized to consist of a diverse pool of macrophage subpopulations, the signals governing the expansion and contraction of these discrete macrophage subsets in normal adipose physiology and disease are not known.

During chronic inflammation, the resolution phase of the inflammatory response fails to occur leading to sustained immune dysregulation. We recently showed that the neuroimmune guidance cue netrin-1 contributes to maladaptive macrophage immune responses in obesity and atherosclerosis by fostering macrophage persistence in tissues. Netrin-1 is a secreted laminin-like molecule initially identified as a guidance cue for axonal growth that directs both chemoattractive and repulsive signaling. These divergent responses to netrin-1 are mediated by distinct receptors on target cells: attraction to netrin-1 is mediated by receptors of the DCC (Deleted in Colorectal Carcinomas) family including DCC and neogenin, whereas repulsion or chemostasis are mediated by members of the UNC5 family and the adenosine A2 B receptor (A2BAR) [29]. Expression of these receptors is not limited to neurons, and we and others have shown that myeloid cells express Unc5b and A2BAR, but not DCC, and can respond to netrin-1 [30–34]. For example, in the setting of transient ischemia, netrin-1 expression by endothelial or epithelial cells is induced via hypoxia-inducible factor 1 alpha (HIF-1α) to maintain barrier function and dampen leukocyte infiltration into tissues [33–36]. Conversely, in obesity and atherosclerosis, expression of netrin-1 and its receptor Unc5b are upregulated in tissue macrophages in response to lipid and oxidative stressors, and can prevent resolution of inflammation by inhibiting macrophage responses to chemokines (e.g., CCL19, CCL21) directing the egress of inflammatory macrophages from tissues and preventing macrophage apoptosis [37–39]. In mice, deletion of Ntn1 in hematopoietic cells by bone marrow transplantation prevented obesity-induced inflammation and improved insulin resistance [37]. We designed the current study to test how targeted deletion of netrin-1 specifically in macrophages alters macrophage accumulation and phenotype in the adipose tissue during obesity.

We show herein that loss of macrophage netrin-1 expression in mice (Ntn1^Δmac^) reduces the inflammation and insulin resistance associated with diet-induced obesity. Flow cytometry analysis revealed a decrease in CD11b^+^CD11c^–^F4/80^+^ and CD11b^+^CD11c^+^F4/80^+^ macrophages in VAT of HFD-fed Ntn1^Δmac^ mice, compared to WT mice. Single cell RNA-seq showed that while HFD-expanded the pool of resident macrophages in WT mice, this subpopulation was specifically depleted in HFD-fed Ntn1^Δmac^ mice. Furthermore, macrophage deficiency of netrin-1 altered the fate and functional trajectory of ATMs in obesity, with the major macrophage subpopulation showing increased expression of genes involved in migration, lipolysis and fatty transport. These findings suggest that targeted deletion of netrin-1 in macrophages improves adipose tissue homeostasis and metabolic dysfunction in obesity.

## MATERIAL AND METHODS

### Mouse Studies

All experimental procedures were done in accordance with the US Department of Agriculture Animal Welfare Act and the US Public Health Service Policy on Humane Care and Use of Laboratory Animals and were approved by the New York University School of Medicine’s Institutional Animal Care and Use Committee (project code: s16–01762, The approval date is 4 May 2019). We generated mice with myeloid-specific deletion of netrin-1 (*Ntn1*^fl/fl^*LysMCre*^+/–^; Ntn1^Δmac^) and littermate control mice (*Ntn1*^fl/fl^*LysMCre*^–/–^; WT) and fed them either a low-fat chow (13% fat kcal, 0% cholesterol; LabDiet #5053) or high fat diet (HFD; 60% fat kcal, 0.3% cholesterol; Dyets #D12492; Research Diets) for 20 weeks. Mice were monitored regularly throughout the study and weights were recorded weekly. Prior to sacrifice, dual-energy X-ray absorptiometry scanning (DEXA; PIXIMus II, Lunar, Madison, WI) was performed to measure the fat and lean mass.

### Tissue Collection and Flow Cytometry

After diet feeding, epididymal VAT was harvested from Ntn1^Δmac^ and WT mice and stored in 10% formalin for tissue sectioning (PPFE, 5 μm), or digested for flow cytometric analysis using the Mouse Adipose Tissue Dissociation Kit (Miltyeni Biotec, Bergisch Gladbach, Germany) and GentleMacs dissociator (Miltenyi biotec, Bergisch Gladbach, Germany). The digested VAT was passed through a 70 μm cell strainer, washed with 1× cold PBS and centrifuged at 350 *g* for 10 min at 4 °C to collect the stromal vascular fraction. Blood collected at sacrifice was lysed with ACK lysis buffer and leukocytes collected. For flow cytometry, cells were stained with Fixable Viability Dye eFluor® 780 (eBioscience, San Diego, USA) for 30 min on ice, blocked with TruStain fcX™ (anti-mouse CD16/32 antibody, San Diego, USA) from Biolegend and then stained using PerCp/Cy5.5 anti-mouse CD45 antibody (Biolegend, 103132, San Diego, USA), Bv650 anti-mouse CD11b antibody (eBioscience, 101239, San Diego, USA), Bv786 anti-mouse F4/80 antibody (Biolegend, 123141, San Diego, USA), Bv570 anti-mouse Ly6C antibody (Biolegend, 128030, San Diego, USA), PE anti-mouse CD11c (Biolegend, 117308, San Diego, USA), Bv421 anti-mouse CD64 antibody (Biolegend, 139309, San Diego, USA), Alexa Fluor 700 anti-mouse CD3 antibody (Biolegend, 100216, San Diego, USA), Bv605 anti-mouse CD4 antibody (Biolegend, 100548), APC anti-mouse CD25 antibody (Biolegend, 101910, San Diego, USA), PE-anti-mouse CD9 (BioLegend, 124806, San Diego, USA) for 30 min on ice. The cells were then fixed and permeabilised using Fixation/Permeabilisation kit (BD Biosciences, San Diego, USA). The cells were then staining for intracellular markers, APC-conjugated anti-netrin-1 (Abcam, ab126729, Cambridge, UK), Bv421-anti mouse Foxp3 (BioLegend, 126419, San Diego, USA), PE-Cy7 anti-mouse Tbet (BioLegend, 644824, San Diego, USA), Bv711 anti-mouse Gata3 (BD Biosciences, 565449, New Jersey, USA) and APC anti-mouse ROR*γδ* antibody (ThermoFisher Scientific, 17-6981-82, Waltham, USA). The cells were then acquired on a LSRII HTS (BD Biosciences, New Jersey, USA) and analyzed with FlowJo V10 (Tree-Star, Ashland, USA).

### Immunohistochemistry

Sections were rehydrated and antigen retrieval was performed using citrate buffer. The sections were then fixed with acetone, permeabilised with 0.1% triton and blocked with 5% BSA. The sections were incubated with primary antibodies, anti-F4/80 for macrophage marker (Abcam, ab6640, Cambridge, UK), anti-netrin-1 (Abcam, ab126729, Cambridge, UK) and anti-UCP1 (Sigma, U6382, St. Louis, USA) followed by respective secondary antibodies. For adipocyte measurements, sections were stained with hematoxylin and eosin. Sections were imaged with a Nikon Eclipse microscope and staining was quantified using image J analysis software (https://fiji.sc/). Adipocyte size was measured using Adiposoft (https://imagej.net/Adiposoft).

### Metabolic Assays

After 18 weeks of diet feeding, mice were starved for 4–5 h and basal glucose was measured using an automatic glucose monitor (Freestyle Lite, Chicago, USA) A glucose tolerance test was performed by i.p. injection of 2 g/kg D-glucose and glucose readings were taken every 15 min as we previously described [37]. For insulin tolerance test, 0.75 mU of insulin was injected intraperitoneally and glucose readings were taken every 15 min as we previously described [37]. Plasma free fatty acids and insulin levels were measured using NEFA from Fujifilm Wako Diagnostics (Tokyo, Japan) and a sensitive insulin ELISA kit (R & D Systems, Minneapolis, USA) respectively. Plasma leptin and adiponectin levels were measured using mouse leptin ELISA kit (R&D Systems, Minneapolis, USA) and mouse adiponectin quantikine ELISA kit (R & D Systems, Minneapolis, USA) respectively.

### qPCR Analysis of Gene Expression

RNA was extracted from either from whole epididymal VAT or F4/80^+^ macrophages from epididymal VAT or peritoneal macrophages of chow and HFD-fed Ntn1^Δmac^ and WT mice using Qiagen Lipid free-RNA extraction kit as previously described [37]. RNA (0.5–1 μg) was reverse transcribed using iScriptTM cDNA Synthesis Kit (Bio-Rad, Hercules, USA) and RT-PCR analysis was conducted using kapa SYBR green Supermix (Kapa Biosystems, Wilmington, USA) and a Mastercycler Realplex (Eppendorf, Hamburg, Germany) Fold change in mRNA expression *Atgl* (Forward primer: 5’CAACGCCACTCACATCTACGG3’, reverse primer: 5’GGACACCTCAATAATGTTGGCAC3’), *Hsl* (Forward primer: 5’CCAGCCTGAGG GCTTACTG3’, reverse primer: 3’CTCCATTGACTGTGACATCTCG5’), *Pparγ* (Forward primer: 5’AACTCTGGGAGATTCTCCTGTTGA3’, reverse primer: 3’TGGTAATTTCTTGTGAAGTGCTCATA5’), *Netrin-1* (Forward primer: 5’CAGCCTGATCCTTGCTCGG3’, reverse primer: 3’GCGGGTTATTGAGGTCGG TG5’), *Pgc1α* (Forward Primer: 5’GAATCAAGCCACTACAGACACCG3’, reverse primer: 3’CATCCCTCTTGAGCCTTTCGTG5’) and *Prdm16* (Forward Primer: 5’GAAGAGCGTGAGTACAAATG3’, reverse primer: 3’AACACCTTGACACAGT TTTC5’) was calculated using the comparative cycle method (ΔΔCt) and normalized to *Hprt* (Forward primer: 5’AAGCCTAAGATGAGCGCAAG3’, reverse primer: 3’GGCCACAGGACTAGAACACC5’), *Gapdh* (Forward primer: 5’AACGACCCCTTCATTGAC3’, reverse primer: 3’TCCACGACATACTCAGCAC5’) and 28S RNA (Forward primer: 5’GCTAAATACCGGCACGAGAC3’, reverse primer: 5’TTCACGCCCTCTTGAACTCT3’) as we previously described [40]. For genotyping, DNA was extracted from peritoneal macrophages or adipose tissue macrophages of Ntn1^Δmac^ and WT mice using Quick DNA extraction kit (Zymo Research, Irvine, USA). PCR for netrin-1 deletion was performed using forward primer: 5’CGTTGAGACAGGACGCTCTTGC3’ and reverse primer 3’GAGGAAGGCAGTGAATGTGTTTC5’.

### Labelling and Tracking of Blood Monocytes

Circulating blood monocytes were labeled by retro-orbital intravenous injection of 1 μm fluorescent red beads (Flouresbrite YG microspheres (Polysciences Inc., Warrington, USA) diluted 1:4 in sterile PBS as described [37,39,41]. The efficiency of bead labelling was verified 24 h later by flow cytometry. One group of mice was harvested after 3 days for baseline measurements of monocyte recruitment, and a second group of mice was harvested 14 days later to measure the number of labeled macrophages remaining in the adipose tissue. After sacrifice, bead-labeled macrophages in the VAT were quantified by flow cytometry analysis.

### Single Cell RNA-Sequencing

Single-cell RNA sequencing was performed on CD45^+^ cells isolated from VAT digested as described above and VAT from 4–5 mice was pooled. Stromal vascular cells were stained for CD45 (anti-CD45 PerCp-Cy5.5, BioLegend, San Diego, USA) and isolated by flow sorting viable (fixable viability dye e780, eBioScience, San Diego, USA) single cells on a Facs Aria II cytometer (BD) equipped with a 100 μm nozzle. Sorted CD45^+^ cells were loaded into single cell gel beads (GEMs) and barcoded with a unique molecular identifier (UMI) using the Single Cell 3’ reagent kit (10× Genomics) and processed as described previously [42]. Subsequently, the sequence libraries containing the full-length, barcoded cDNA were generated and sequenced on a NovaSeq 6000 (Illumina, San Diego, USA) in a dual paired-end sequencing. The data was demultiplexed, QC checked and aligned to the mouse genome using the Cell Ranger Single Cell Software Suite v2.2, as described at https://support.10xgenomics.com/single-cell-gene-expression/software/pipelines/latest/what-is-cell-ranger. Briefly, using *cellranger mkfastq* and *cellranger count*, FASTQ files were generated and aligned to the mm10 genome, sequencing reads were filtered by base-calling quality scores, and then cell barcodes and UMIs were assigned to each read in the FASTQ files. The filtered gene expression matrices were then used for downstream analyses. Outlier cells were removed based on the number of genes expressed and the percent of expressed genes coming from the mitochondria, as outliers in these metrics are likely to represent either multiple captured cells or cells undergoing apoptosis. Using the R package Seurat, the samples were then merged, canonical correlation analysis (CCA) was performed, and the canonical correlation vectors (CCs) were aligned, as described by Butler *et al.* [43]. Using the aligned CCs, Louvain clusters were found and tSNE dimension reduction was performed.

### Cluster Annotation and Differential Gene Expression

To assign clusters and individual cells to main cell types, we used the R package SingleR [44] and default parameters, using Immgen as the reference dataset and the parameter do.main.types = T. This resulted in the most likely main cell type being assigned to either each cluster, based on the average expression profile of the cluster, or each individual cell, using the expression profile of the cell. We used Seurat [43] to identify differentially expressed genes by sample for each cluster, using the Wilcoxon test to generate *p*-values. To calculate log fold-change (logFC) values and *p*-values for all variable genes for each cluster, we used the following parameters: logfc.threshold = −Inf, min.pct = 0, min.cells.gene = 0, min.cells.group = 0, genes.use = my.seurat@var.genes. We used the R package ClusterProfiler [45] to look for enriched functions in the marker genes for each cluster, as well as differentially expressed genes by sample. Specifically, we used the enrichGO and enrichKEGG functions to look for terms that were enriched in particular clusters, and the compareClusters function to look for terms that showed differential enrichment across clusters. In addition, we used the online tool Gorilla [46] to cross-validate and investigate enrichment of GO terms for specific clusters and differential expression analyses.

### Pseudotime and Branched Gene Expression Analysis

To analyze the trajectories of cells identified as monocytes and macrophages in our single cell RNA-seq, we used the Monocle algorithm [47]. Briefly, Monocle was used to estimate size factors, dispersion, and differential gene expression of the cells, and the top 1000 most differentially expressed genes were used to order the cells in pseudotime. We defined the branch with the largest proportion of monocytes as the root state of the tree, and plotted the trajectory of obese WT *vs* Ntn1^Δmac^ macrophages along the same pseudotime trajectory. We used the BEAM feature of Monocle to define genes that show significantly divergent expression across each branch point in the pseudotime analysis.

### Targeted LC-MS/MS Metabololipidomics Analysis

Mass spectrometry-based lipidomics were used to map the pro-inflammatory and pro-resolving lipidome in VAT as previously described [48]. VAT from chow and HFD-fed Ntn1^Δmac^ and WT (*n* = 5) was snap frozen in liquid nitrogen. Lipid mediators were extracted using solid phase C18 columns and elution in methyl formate. Identification and quantification of lipid mediators by LC-MS/MS was facilitated by internal deuterated standards and external standard curves for each mediator, as we previously described [48].

### Statistical Analyses

The difference between two groups was analyzed by Student’s *t*-test or for multiple comparisons, by one-way analysis of variance, followed by Sidak’s multiple comparison test assuming Gaussian distribution and by non-parametric Kruskal-Wallis test. *P* values of less than 0.05 were considered significant. Analyses were performed using GraphPad Prism8 (GraphPad Software, Version 8.02).

## RESULTS

### Macrophage-Specific Deficiency of Netrin-1 Improves Metabolic Function in Obesity

**Figure 1.**
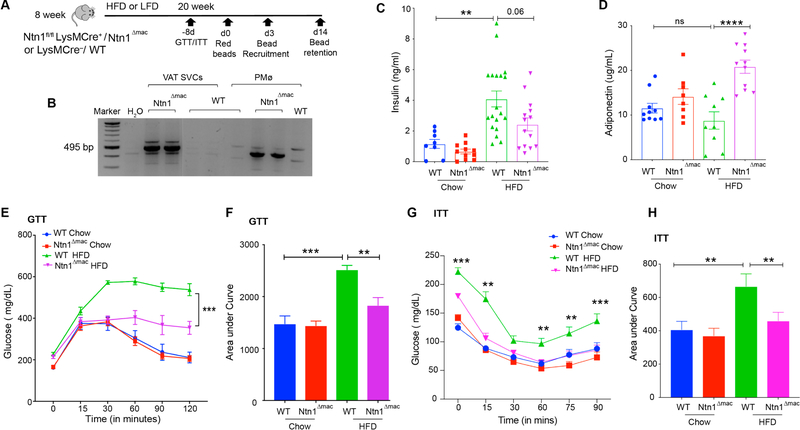
Macrophage-specific deficiency of netrin-1 improves metabolic function in obesity. Ntn1^Δmac^ or WT mice were fed low fat diet (LFD) or high fat diet (HFD) for 20 weeks to establish obesity. (**A**) Experimental approach showing times of glucose and insulin tolerance tests (GTT, ITT), and fluorescent bead injection for measurements of monocyte recruitment and macrophage retention. (**B**) Confirmation of netrin-1 deletion in DNA isolated from SVCs of VAT and peritoneal macrophages from Ntn1^Δmac^ or WT mice (control). Deletion of netrin-1 is indicated by a band of 495 bp. (**C–D**) Plasma insulin (C) and adiponectin (D) levels in WT and Ntn1^Δmac^ mice fed chow and HFD. *n* = 10–15 mice per group. (**E–F**) Glucose tolerance test on mice of the indicated genotype showing (E) plasma glucose level at the indicated times after glucose injection (i.p.; 2 g/kg) and (F) area under curve. (**G–H)** Insulin tolerance test on mice of the indicated genotype showing (G) plasma glucose level at the indicated times after insulin injection (i.p., 0.75 mU/kg) on fasted mice, and (H) area under curve. *n*= 20–24 mice per group. Data are the mean ± SEM; Statistical analyses were performed by non-parametric Kruskal-Wallis test (panel 1C) or one-way ANOVA with post-hoc Sidak’s test (panel 1D-1H). *p < 0.05, **p < 0.01, ***p < 0.001, ****p < 0.0001.

Previous studies from our group showed that hematopoietic expression of netrin-1 promotes adipose tissue inflammation and metabolic dysfunction [37]. Our studies suggested that adipose tissue macrophages that accumulate with obesity are the source of netrin-1 that drives macrophage accrual in adipose tissue and contribute to insulin resistance. To definitively test this, we generated mice with myeloid-specific deletion of Netrin-1 by crossing Ntn1^fl/fl^ mice with mice expressing the Cre gene under control of the LysM promoter. The resultant LysMCre^+/–^Ntn1^fl/fl^ (Ntn1^Δmac^) and control LysMCre^–/–^ Ntn1^fl/fl^ (WT) mice were placed on a low-fat chow diet or a high fat diet (HFD) to establish diet-induced obesity for 20 weeks, and adipose inflammation and metabolic function were assessed (Figure 1A). We confirmed efficient deletion of Netrin-1 in peritoneal macrophages and stromal vascular cells isolated from epididymal VAT in Ntn1^Δmac^ mice, which generates a PCR-amplifiable fragment of 495 bp upon Netrin-1 deletion (Figure 1B). To assess whether the selective deletion of netrin-1 in macrophages alters obesity-associated adipose tissue inflammation and insulin resistance, we measured fasting insulin levels and performed glucose and insulin tolerance tests (GTT and ITT) in Ntn1^Δmac^ and WT mice 18 weeks after chow or HFD feeding. While plasma insulin levels were similar in mice of both genotypes fed chow diet, upon HFD feeding WT mice showed markedly higher fasting insulin levels than Ntn1^Δmac^ mice (Figure 1C). Obese Ntn1^Δmac^ mice showed improved glucose tolerance after intraperitoneal injection of a glucose bolus compared to obese WT mice (Figure 1E–F). Furthermore, obese Ntn1^Δmac^ mice were more insulin sensitive than obese WT mice as measured by an insulin tolerance test (Figure 1G–H). Finally, we observed an increase in plasma levels of the anti-inflammatory and insulin-sensitizing protein adiponectin in HFD-fed Ntn1^Δmac^ compared to WT mice (Figure 1D), consistent with improved insulin sensitivity.

### Ntn1^Δmac^ Mice are Partially Protected from Diet-Induced Obesity

**Figure 2.**
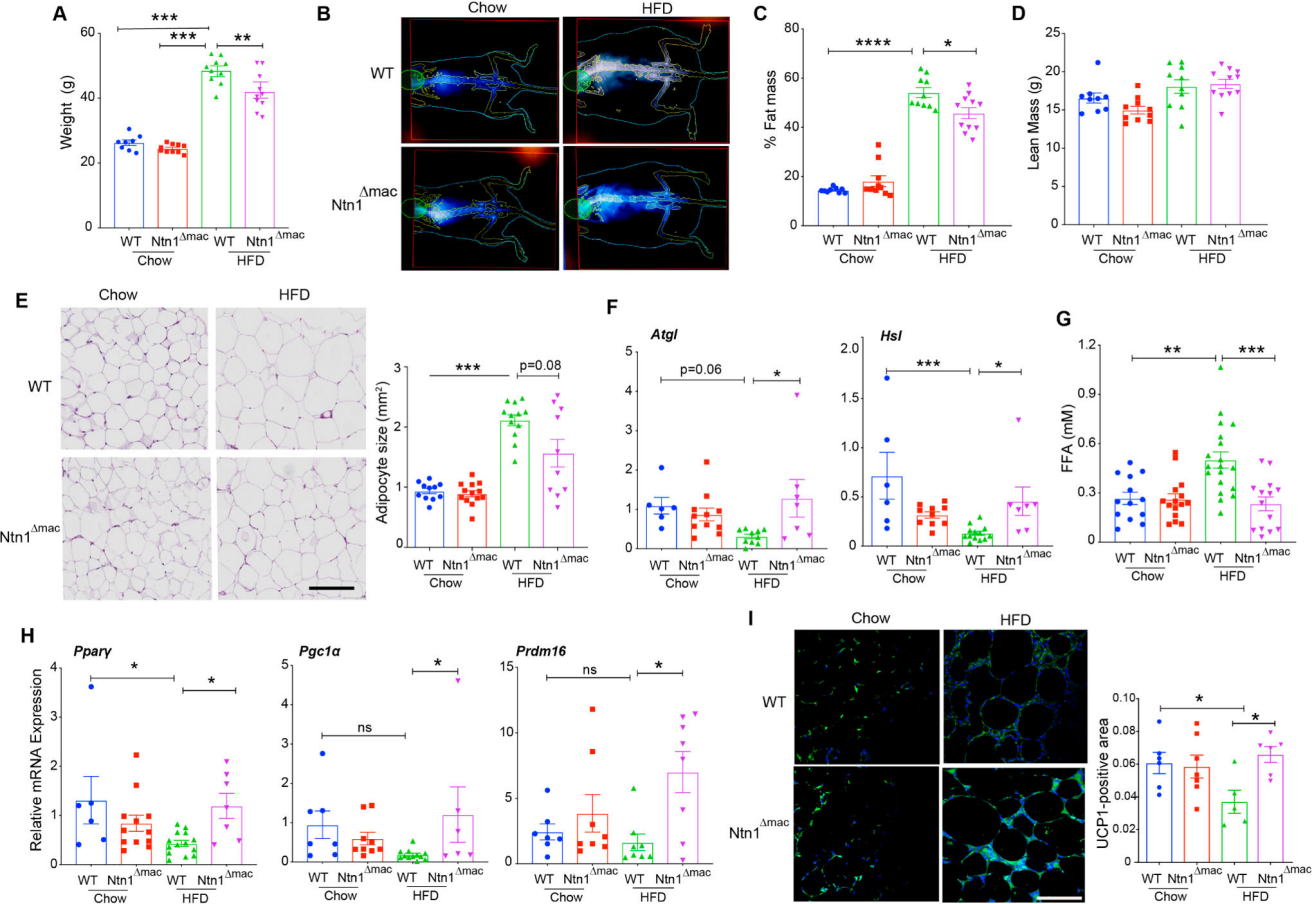
Ntn1^Δmac^ mice are partially protected from diet-induced obesity. (**A**) Weight of Ntn1^Δmac^ or WT mice fed chow or HFD for 20 weeks. (**B**) Representative DEXA scan images and calculation of (**C**) fat mass and (**D**) lean mass composition of chow and HFD-fed Ntn1^Δmac^ or WT mice. *n* = 10 mice/group. (**E**) Representative images of H & E stained VAT sections of WT and Ntn1^Δmac^ mice fed chow or HFD. Scale bar = 500 uM. Quantification of adipocyte size shown at right. *n* = 10 mice per group. (**F**) qPCR analysis of *Atgl* and *Hsl* mRNA in VAT from WT or Ntn1^Δmac^ mice. *n* = 6–9 mice per group. (**G**) Plasma levels of free fatty acids in WT or Ntn1^Δmac^ mice. *n* = 15–20 mice per group. (**H**) qPCR analysis of *Pparg*, *Pgc1a* and *Prdm1*6 mRNA in VAT from WT or Ntn1^Δmac^ mice. *n* = 6–9 mice per group. (**I)** Representative images of UCP1 immunostaining in VAT sections of WT and Ntn1^Δmac^ mice fed chow or HFD. Scale bar = 100 uM. Quantification of UCP1-positive area is shown at right. Data are the mean ± SEM; Statistical analyses were performed by non-parametric Kruskal-Wallis test (panel 2E, 2H) or one-way ANOVA with post-hoc Sidak’s test (panel 2A, 2C, 2D, 2F, 2I). *p < 0.05, **p < 0.01, ***p < 0.001, ****p < 0.0001.

To understand the mechanisms underlying improved insulin sensitivity in obese mice with macrophage-specific deletion of netrin-1, we measured weight gain and adiposity in WT and Ntn1^Δmac^ mice fed chow and HFD. We previously reported that deficiency of netrin-1 in all bone marrow derived cells did not affect body mass or HFD-induced weight gain in mice. Interestingly, although both WT and Ntn1^Δmac^ mice gained weight upon HFD-feeding, Ntn1^Δmac^ mice weighed 15% less than WT mice after 20 weeks of HFD (Figure 2A). We observed no genotype-dependent difference in total body mass in mice fed chow diet (Figure 2A). Using DEXA scanning, we confirmed that both WT and Ntn1^Δmac^ mice became obese upon HFD feeding, with adiposity between 40% and 50% (Figure 2B–C), yet fat mass was unexpectedly lower in Ntn1^Δmac^ mice (15.2 g *vs* 21.3 g). By contrast, we observed no difference in lean body mass (Figure 2D), food consumption (Supplementary Figure S2A) or leptin levels (Supplementary Figure S2B) between WT and Ntn1^Δmac^ mice fed HFD suggesting that macrophage-specific deletion of netrin-1 partly attenuated the development of obesity. Consistent with this, adipocyte size, while similar in lean mice of both genotypes, was significantly lower in Ntn1^Δmac^ mice than WT mice fed HFD (Figure 2E).

During obesity, the increased triacylglycerol (TAG) storage in adipocytes and expansion of adipose tissue is thought to be a beneficial, adaptive response that limits ectopic lipid deposition in tissues and lipotoxicity. The canonical release of lipid from adipocytes is mediated through the sequential actions of the neutral lipases adipose tissue triglyceride lipase (ATGL) and hormone-sensitive lipase (HSL). To investigate whether differences in the expression of these lipases was associated with the reduced adipocyte mass in Ntn1^Δmac^ mice, we measured mRNA expression of ATGL and HSL (Lipe). We observed no genotype-specific differences in *Atgl* and *Hsl* mRNA in lean mice, and as expected the expression of these lipases decreased in WT mice fed HFD (Figure 2F). However, expression of *Atgl* and *Hsl* mRNA was higher in HFD-fed Ntn1^Δmac^ mice compared to HFD-fed WT, with expression levels similar to lean mice (Figure 2F), suggesting that efficient TAG lipolysis occurs in the absence of macrophage derived netrin-1. Despite this, we found that plasma levels of non-esterified fatty acid were reduced in obese Ntn1^Δmac^ mice compared to WT mice, with circulating FFA levels in HFD-fed Ntn1^Δmac^ mice similar to those observed in lean mice (Figure 2G). As levels of FFAs are determined by the balance of TAG lipolysis, fatty acid uptake and β-oxidation, we measured the expression of genes involved in the regulation of fatty acid uptake and mitochondrial respiration. We observed an increase in *Pparg* and *Ppargc1a* (PGC1α) mRNA levels in VAT from Ntn1^Δmac^ mice compared to WT mice fed HFD (Figure 2H). Furthermore, VAT of Ntn1^Δmac^ mice showed higher mRNA levels of *Prdm16* (Figure 2H), a transcriptional coregulator of thermogenic gene programs, as well as increased immunostaining for the mitochondrial uncoupling protein UCP-1 (Figure 2I), suggestive of VAT “beiging”. Together, these data suggest that in the absence of netrin-1, there is an increase in fatty acid oxidation gene programs and energy expenditure through uncoupled respiration in VAT.

### Deletion of Macrophage-Derived Netrin-1 Reduces ATM Accumulation

**Figure 3.**
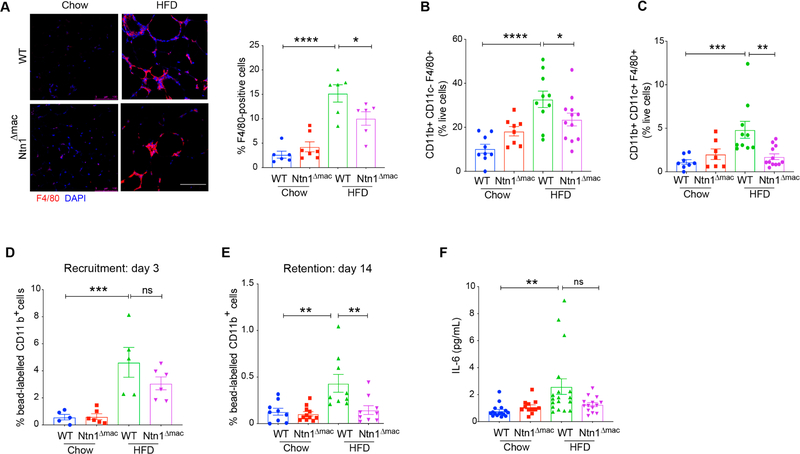
Macrophage netrin-1 deficiency reduces adipose tissue macrophage accumulation in obesity. (**A**) Representative images of F4/80^+^ stained macrophages of VAT sections of WT and Ntn1^Δmac^ mice fed chow and HFD. Scale bar = 100 uM. Quantification of F4/80^+^ macrophages shown at right. (**B**) Quantification of CD11b^+^CD11c^−^F4/80^+^ and (**C**) CD11b^+^CD11c^+^F4/80^+^ macrophages in VAT by flow cytometry analysis. (**D–E**) Recruitment of (D) bead-labeled monocyte recruitment 3 days after pulse-labeling, and (E) retention of bead-labeled macrophages 14 days after pulse-labeling, quantified by flow cytometric analysis of VAT. (**F**) Plasma levels of IL-6 in WT and Ntn1^Δmac^ mice fed chow and HFD. Data are the mean ± SEM; Statistical analyses were performed by non-parametric Kruskal-Wallis test (panel 3F) or one-way ANOVA with post-hoc Sidak’s test (panel 3A–E). *p < 0.05, **p < 0.01, ***p < 0.001 ****p < 0.0001.

During obesity, monocyte-derived macrophages accumulate in VAT and are a source of inflammatory mediators that contribute to insulin resistance. In addition, adipose tissue macrophages (ATMs) are thought to buffer the increased concentration of lipids in the expanding adipose tissue by participating in lipid uptake, trafficking and storage. Previous studies from our group showed that netrin-1 expression in obese adipose tissue contributes to the accumulation of ATMs by inducing macrophage chemostasis and inhibiting macrophage egress from the VAT. Consistent with ATMs being the primary source of netrin-1 in obese adipose tissue, we find that macrophage-specific deletion of netrin-1 reduced macrophage accumulation in VAT in HFD-fed mice by 35%, as measured by F4/80 staining (Figure 3A). Obese adipose tissue has been shown to contain both CD11b^+^CD11c^−^ and CD11b^+^CD11c^+^ F4/80 macrophage [12,49,50]; with the latter population being linked to increased inflammation and IR [11]. Flow cytometric analysis of CD45^+^ cells isolated from digested VAT showed that the decrease in F4/80^+^ macrophages was due to reduced levels of both CD11b^+^CD11c^−^ and CD11b^+^CD11c^+^ macrophages in Ntn1^Δmac^ compared to WT HFD-fed mice (Figure 3B–C). By contrast, no genotype-specific difference in F4/80^+^ macrophage accumulation was observed in chow fed mice, consistent findings of total hematopoietic deficiency of Ntn1 [37]. Similar results were found using the macrophage marker CD64 (Supplementary Figure S3A–D). In addition, we observed a change in the balance of CD4^+^ T cell subsets in HFD-fed Ntn1^Δmac^ mice with an increase in anti-inflammatory Th2 and regulatory T cells (Tregs) compared to WT mice fed HFD (Supplementary Figure S3E–I).

To investigate if the reduced macrophage content in HFD-fed Ntn1^Δmac^ mice was due to reduced recruitment of monocytes or reduced retention of macrophages in the VAT, we traced monocyte-macrophage dynamics using a bead-labeling technique as previously described [37]. Consistent with previous studies [37], HFD-feeding increased the recruitment of labeled monocytes into VAT by approximately 4-fold over chow fed mice, and macrophage expression of netrin-1 did not significantly alter monocyte recruitment in either chow or HFD-fed mice (Figure 3D). However, analysis of bead-labeled macrophages retained in the VAT 14 days later showed that the VAT of Ntn1^Δmac^ mice contained 60% fewer macrophages than WT mice fed HFD (Figure 3E). Consistent with reduced macrophage retention and adipose inflammation in obese Ntn1^Δmac^ mice, we observed lower plasma levels of interleukin-6 (IL-6) in HFD-fed Ntn1^Δmac^ mice compared WT mice (Figure 3F).

### Single Cell RNA-Sequencing Reveals Altered Macrophage Subsets in VAT of Ntn1^Δmac^ Mice

**Figure 4.**
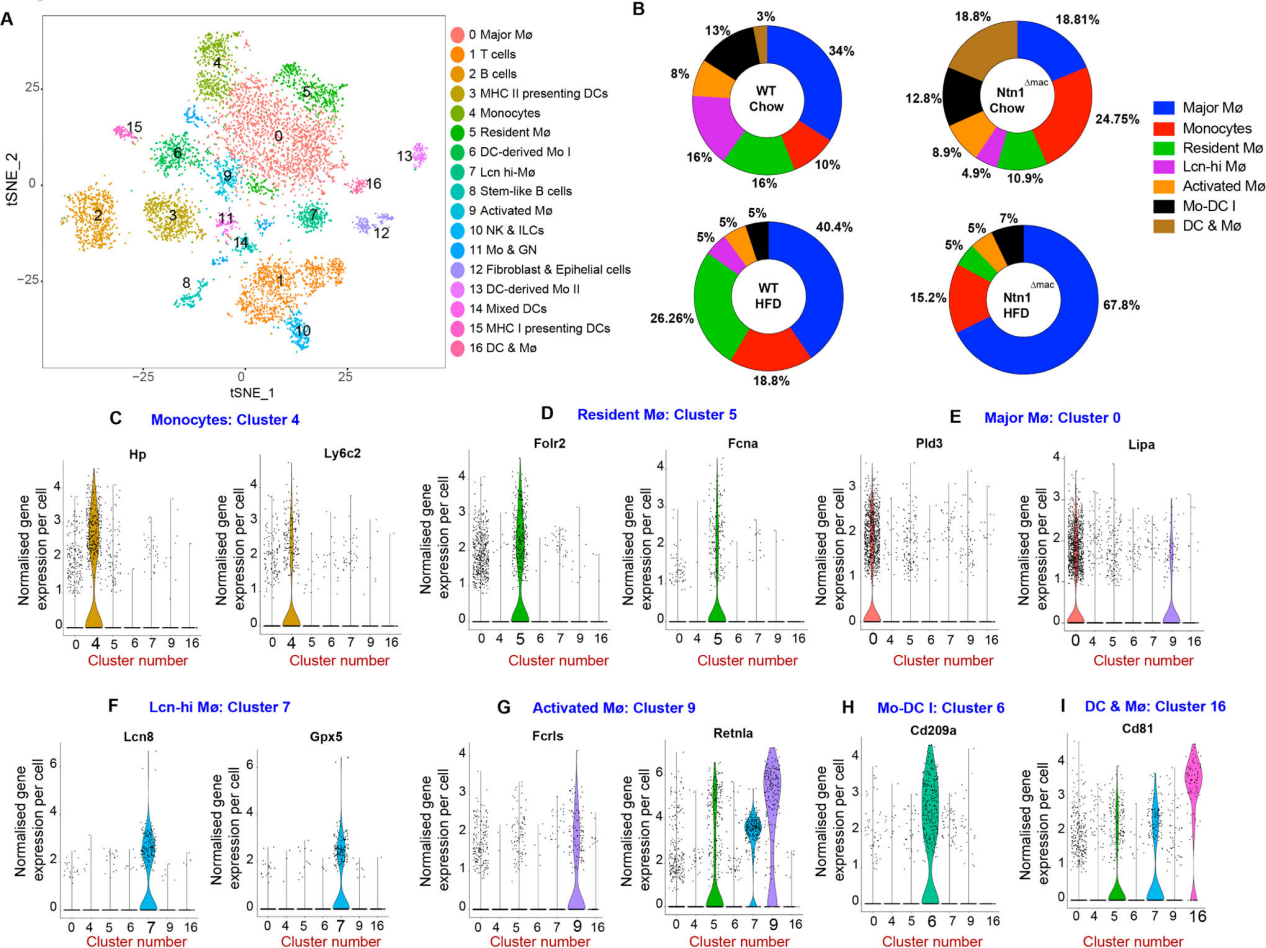
Single cell RNA-sequencing reveals macrophage heterogeneity in adipose tissue. (**A**) Diagram of t-stochastic neighbor embedding (t-SNE) map displaying the distribution of CD45^+^ cells from epididymal VAT of WT and Ntn1^Δmac^ mice fed either chow or HFD (*n* = 4–5 mice; pooled). Colors correspond to clustering of immune cell populations. (**B**) Distribution of monocyte and macrophage clusters in VAT of WT and Ntn1^Δmac^ mice fed either chow or HFD feeding. Data are represented as a percentage of the total monocyte/macrophage pool. (**C–I**) Expression of principal cluster specific markers in the 7 monocyte/macrophage clusters shown as normalized gene expression per cell.

To understand how netrin-1 alters the phenotype of macrophages in the VAT during obesity, we performed single cell RNA-sequencing (scRNA-seq) of CD45^+^ cells isolated from VAT of WT and Ntn1^Δmac^ mice fed HFD, using the 10× Genomics platform. We analyzed 2740 and 1320 cells from WT and Ntn1^Δmac^ mice fed chow, and 1697 and 2765 cells from WT and Ntn1^Δmac^ mice HFD mice. Unbiased hierarchical clustering revealed 17 distinct immune cell clusters in the VAT of obese mice, which were visualized using multicore t-stochastic neighbor embedding (t-SNE) algorithm (Figure 4A). To characterize the main cell types of origin for the clusters, we used SingleR, which leverages the Immgen database to characterize cells by their closest match in an unsupervised manner [51]. The top 5 genes with the highest differential expression for a given cluster as compared to all other cells in the dataset are shown in Supplementary Figure S4. While most immune cell subsets fell into a single cluster (T cells, NK cells) or 2 clusters (B cells, granulocytes, dendritic cells), we identified 7 monocyte or macrophages clusters suggesting considerable diversity among adipose tissue macrophages (Figure 4A). The immune cell clusters identified showed high correlation with a recent independent single cell analysis of immune cells in VAT of lean and obese mice from our groups [52], as shown in the overlaid t-SNE plots (Supplementary Figure S5B).

To investigate whether the composition of monocytes and macrophages in VAT were affected upon myeloid deficiency of netrin-1, we assessed the proportions of myeloid cell subpopulations in chow and HFD-fed WT and Ntn1^Δmac^ mice. As previously reported [7,8], we observed an increase in *Folr2* and *Fcna*-expressing resident macrophages (cluster 5) upon HFD-feeding of WT mice compared to lean controls (26% *vs* 16%)(Figure 4B,D). GO and KEGG Gene Pathway Enrichment analyses indicate functions for this macrophage cluster in scavenger receptor activity, endocytosis, and cytokine and chemokine activity (Supplementary Figure S6). Notably, this resident VAT macrophage pool was markedly reduced in HFD-fed mice lacking macrophage expression of netrin-1, dropping from 26% of monocyte-macrophages in obese WT mice to only 5% in obese Ntn1^Δmac^ (Figure 4B,D). Similarly, we observed a depletion of Lcn8-expressing macrophages (cluster 7) in VAT of obese Ntn1^Δmac^ compared to WT mice (Figure 4B,F). Gene pathway enrichment analyses indicate functions for Lcn-hi macrophages in innate immune activation (cell adhesion, Toll-like receptor binding, endocytosis, TNF signaling, arachidonic acid metabolism), fatty acid binding and antigen processing and presentation, as well as oxidant stress (Figure 4F, Supplementary Figure S3). By contrast, we observed an expansion of the major macrophage cluster associated with obesity (cluster 0), which is characterized by high expression of *Pld3* and *Lipa*, and oxidant stress, lipid metabolism, PPAR signaling and phagolysosomal functions in VAT of obese Ntn1^Δmac^ compared to WT mice (67% *vs* 40%, Figure 4B,E).

**Figure 5.**
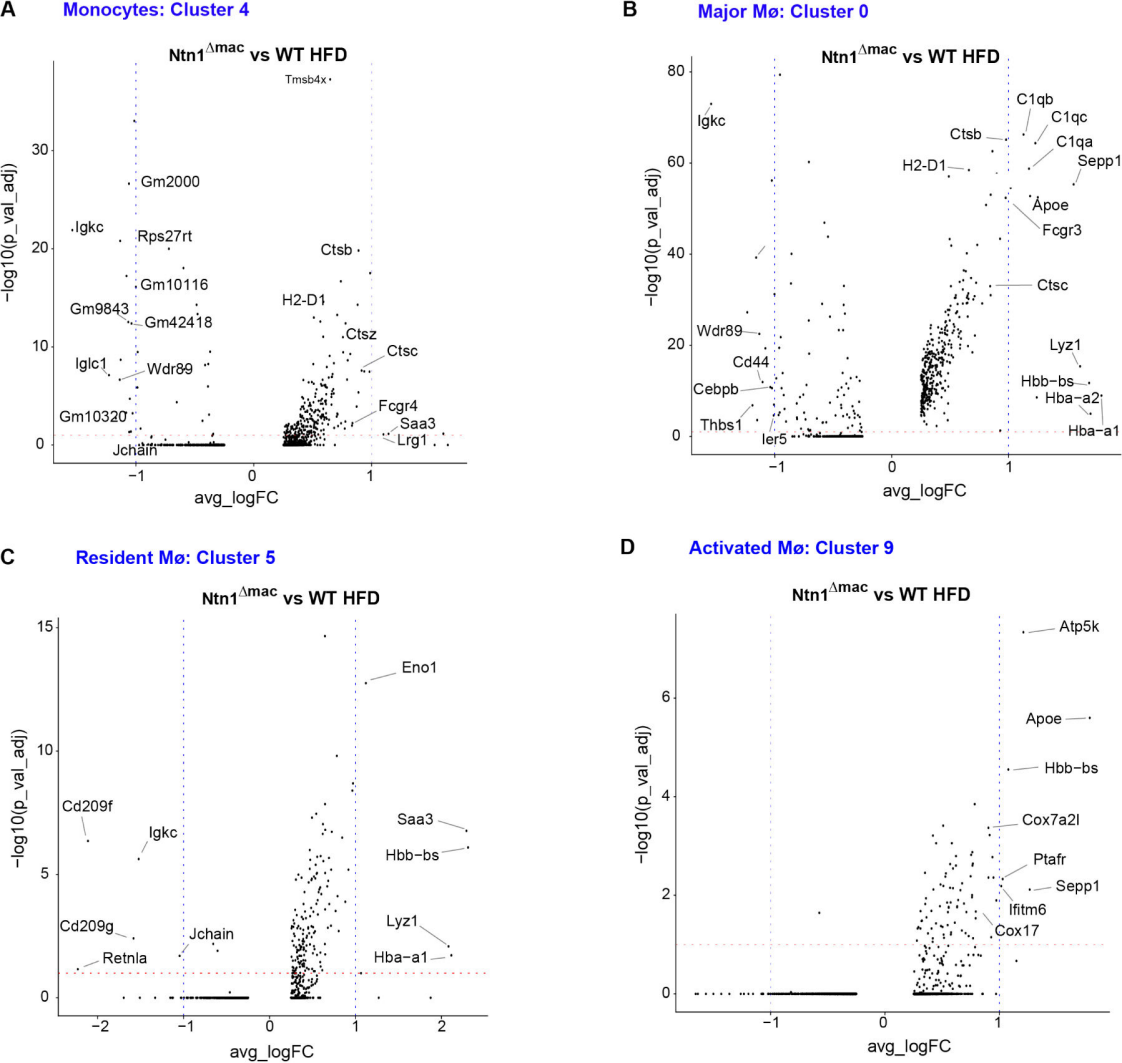
Myeloid-specific deletion of netrin-1 alters the gene expression signature of monocytes and macrophages in the obese adipose tissue. Volcano plots of differentially expressed genes in monocyte (**A**) and macrophage, (**B–D**) clusters from VAT of HFD-fed WT and Ntn1^Δmac^ mice. Statistical analysis was performed by Wilcox-analysis test.

To understand how deletion of netrin-1 altered the phenotype of ATMs, we performed differential gene expression analysis of the major monocyte and macrophage clusters, including monocytes (cluster 4), resident macrophages (cluster 5), the major macrophage cluster in obesity (cluster 0), and activated macrophages (cluster 9)(Figure 5). We found that netrin-1-deficient monocytes had higher expression of genes involved in actin polymerization (*Tmsb4x*), innate immune response (the lysosomal proteases *Ctsz*, *Ctsb* and *Ctsc*, Fc receptor *Fcgr4*, and *Saa3*) and antigen presentation (*H2-D1*), as compared to WT macrophages of the same cluster (Figure 5A). Furthermore, Ntn1^Δmac^ cells belonging to the major macrophage cluster observed in obese VAT had higher expression of genes involved in the innate immune response (e.g., complement components *C1qa*, *C1qb* and *C1qc*, the Fc receptor *Fcgr3*/CD16, and the lysosomal proteases *Ctsb*, *Ctsc* and *Lyz1*) and the M2 macrophage marker *Sepp1*, while they had lower expression of genes involved in cell-cell or cell-matrix interactions (e.g., *Thsb1*, *Cd44*) and *Cebpb*, an important transcriptional regulator of immune and inflammatory response genes, as compared to major macrophages of obese WT mice (Figure 5B). Netrin-1 deficient resident macrophage had higher expression of the glycolytic enzyme *Eno1*, the hydrolase *Lyz1*, the acute phase protein *Saa3*, and M2 macrophage marker *Sepp*1, while they had lower expression of the M2-like macrophage marker *Retnla* and the C-type lectin *Cd209* (DC-SIGN), compared to WT resident macrophages from obese mice (Figure 5C). Finally, Ntn1^Δmac^ cells belonging to activated macrophage cluster had higher expression of mitochondrial genes involved in oxidative phosphorylation (e.g., *Atp5k, Cox7a2I, Cox17*) and *Apoe* compared to WT activated macrophages in obese mice (Figure 5D). Together, these data suggest that netrin-1 expression by macrophages alters both the subtype of macrophages that accumulate in adipose tissue with obesity, and their gene expression profiles.

### Netrin-1 Alters Macrophage Fate and Function in Obese Adipose Tissue

**Figure 6.**
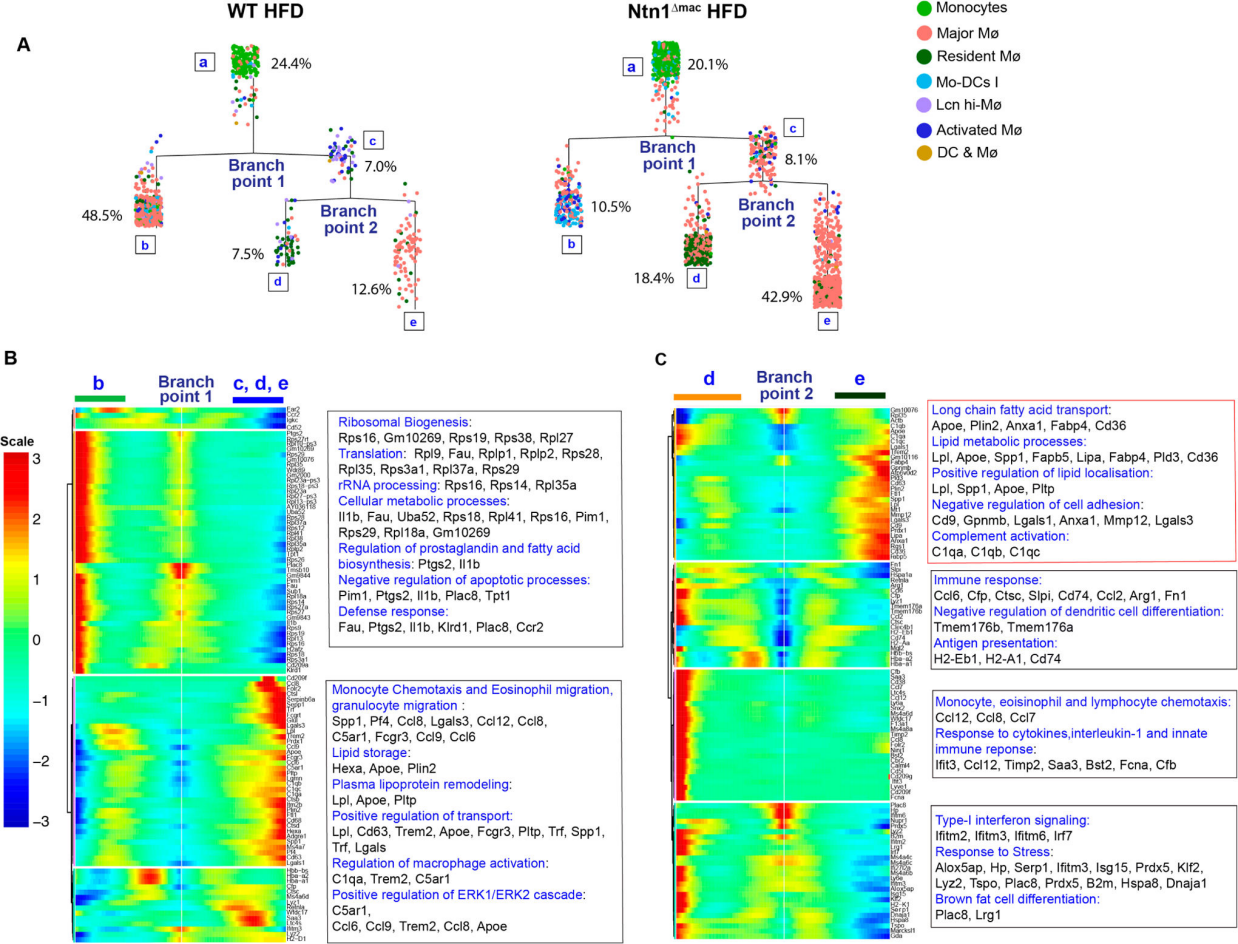
Pseudotime analysis shows distinct trajectories of ATMs in the presence and absence of netrin-1. (**A**) Pseudotime analysis of single cell RNA-sequencing of monocyte and macrophage clusters in VAT of HFD-fed WT and Ntn1^Δmac^ mice. The Monocle algorithm was used to generate a minimum spanning tree, with monocytes defined as the root population. The major branch points and percentage of cells in each phase (a–e) are indicated on the tree. (**B–C**) Heat map of differential expression of cells at (B) branch point 1 for phase b *vs* phases c, d & e, and (C) branch point 2 for phase d *vs* e. Top KEGG and GO terms of the differentially expressed genes are indicated at right of heatmaps.

During our scRNA-seq analysis, we noticed that the majority of the macrophage clusters defined were contiguous, apart from clusters 7 (*Lcn-*hi macrophage) and 16 (dendritic cell and macrophages), suggesting a spectrum of related macrophage activation states (Figure 4). To further investigate this, we used the Monocle algorithm to order single-cell expression profiles in “pseudotime”, which provides a quantitative measure of progress through biological processes such as differentiation or cell fate decisions [47]. Monocle constructs a minimum spanning tree from the single cell transcriptional profiles and defines the longest path through the tree to produce a trajectory of a cell’s progress through cell fate decisions. As shown in Figure 6, Monocle can then reconstruct branched biological processes, which may arise when a cell makes fate decisions that alter its trajectory, and then orders and connects these subtrajectories to the main trajectory. Using the monocyte cluster as the origination point “a”, Monocle decomposed the transcriptomes of WT and Ntn1^Δmac^ macrophages into a two-phase pseudotime trajectory. In WT HFD-fed mice, 24.4% of cells clustered with monocytes (phase a), with the majority of macrophages (48.5%) branching to a state “b” characterized by high demand for protein synthesis (increased expression of genes of ribosomal biogenesis, RNA translation and processing), metabolic processes, and inflammatory mediator gene expression (Figure 6A,B). A smaller proportion of macrophages followed an alternative trajectory (state “c” and its sub-trajectories), with 7.5% of cells branching to a state “d” characterized by high expression of migration and immune response genes and 12.6% of cells branching to a state “e” characterized by high expression of lipid handling and transport genes (Figure 6A,C). Notably, we found that macrophage trajectories in mice lacking macrophage expression of netrin-1 were markedly different. While the proportion of cells that clustered with monocytes was similar in Ntn1^Δmac^ and WT mice (20.1% *vs* 24.4%), only a 10.5% of Ntn1^Δmac^ cells transitioned to the predominant state “b” assumed by WT macrophages, which was characterized by high ribosomal biogenesis, RNA translation and processing, metabolic processes, and inflammatory mediator gene expression (Figure 6A,B). By contrast, the majority of Ntn1^Δmac^ macrophages (70%) proceeded down an alternate path to state “c” and its subtrajectories, with 18.4% of macrophages branching to a state of high migratory and immune response gene expression “d” and 42.9% of macrophages assuming a state of high expression of fatty acid transport, lipid metabolism, and negative regulation of cell adhesion genes “e”. These findings suggest that deletion of netrin-1 alters the fate of adipose tissue macrophages, with the majority of macrophages activating a program of genes specialized in lipid handling, including high expression of genes involved in fatty acid uptake and intracellular transport (*Cd36*, *Fabp4*, *Fabp5*), lipid droplet formation (*Plin2*) and lipolysis (*Lipa*), and regulation of lipid localization (*Apoe*, *Pltp*, *Lpl*).

### Macrophage-Netrin-1 Deletion Reduces Pro-inflammatory Lipid Mediators in VAT

**Figure 7.**
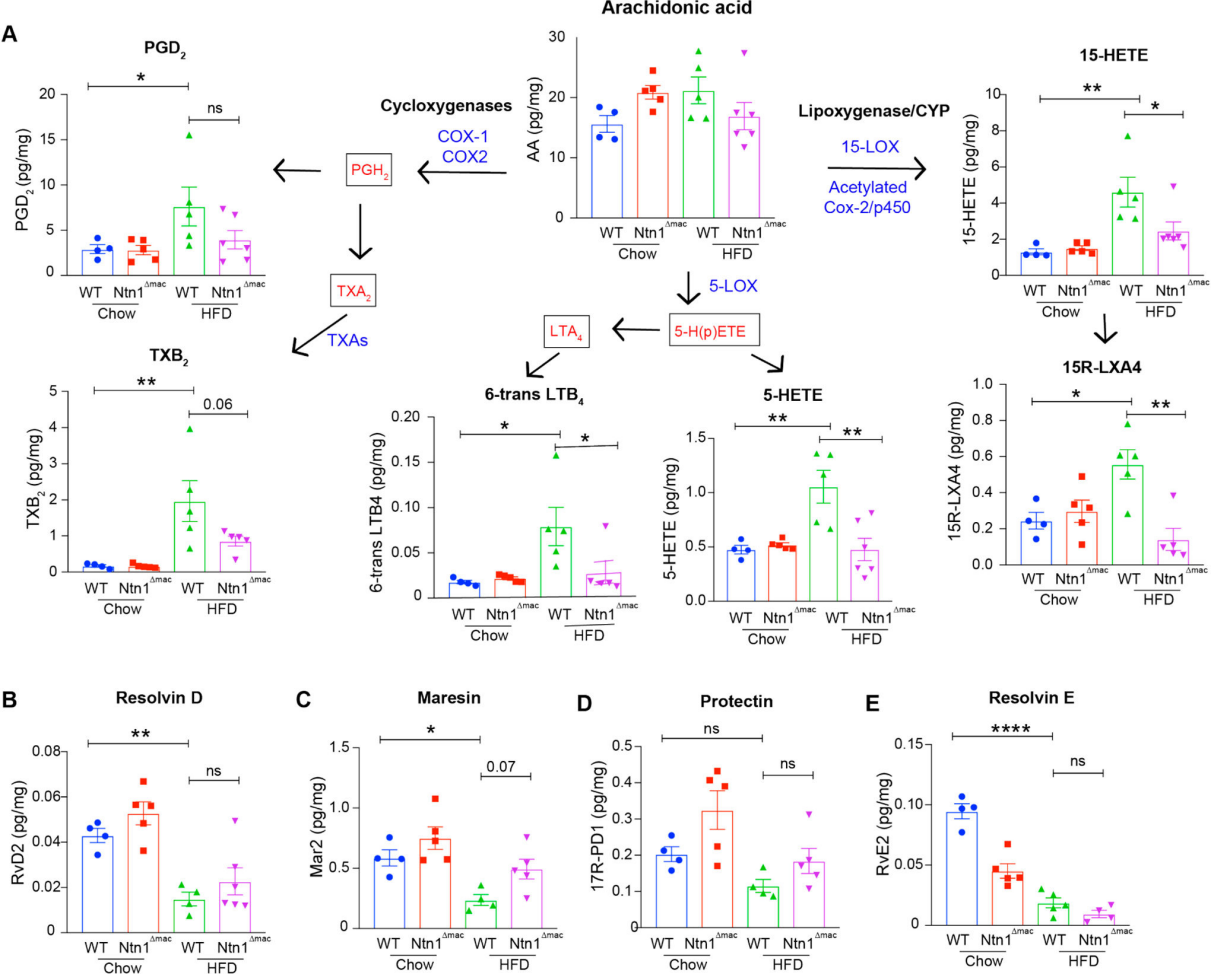
Macrophage deficiency of netrin-1 reduces the levels of pro-inflammatory bioactive eicosanoids derived from arachidonic acid. (**A**) LC-MS/MS analysis of cyclooxygenase and lipoxygenase derivatives of arachidonic acid in VAT of chow and HFD-fed WT and Ntn1^Δmac^ mice. *n* = 4–5 mice/group. (**B–E**) LC-MS/MS analysis of pro-resolving lipid mediators, including (B) resolving D2, (C) Maresin 2, (D)17-RProtectin D1and (E) resolvin E2. (RvD2, MaR2, 17R-PD1 and RvE2). Data are the mean ± SEM; Statistical analyses were performed by one-way ANOVA with post-hoc Sidak’s test. *p < 0.05, **p < 0.01, ***p < 0.001, ****p < 0.0001.

The pseudotime resolution of transcriptional dynamics in WT and Ntn1^Δmac^ macrophages from HFD-fed mice revealed differential gene expression of enzymes involved in the production of pro-inflammatory lipid mediators derived from arachidonic acid (e.g., *Ltc4s, alox5ap, Ptgs2, Ptgds*). Furthermore, netrin-1 deficiency was found to result in a loss of *Lcn*-hi macrophages associated with arachidonic acid metabolism in the VAT of obese mice (Figure 4B). As pro-inflammatory metabolites of arachidonic acid have been implicated in sustaining adipose tissue inflammation and insulin resistance in obesity [53–55], we performed targeted LC-MS/MS metabololipidomic analyses of VAT of WT and Ntn1^Δmac^ to measure bioactive mediators generated from arachidonic acid, docosahexaenoic acid (DHA) and eicosapentaenoic acid (EPA), including pro-inflammatory leukotrienes and prostaglandins, as well as specialized pro-resolving mediators (e.g., resolvins, lipoxins). Consistent with previous reports [53–56] we observed that HFD-feeding of WT mice increased the accumulation of pro-inflammatory metabolites of arachidonic acid in VAT, including those derived through the actions of cyclooxygenases (prostaglandin D_2_, thromboxane B_2_), 5-lipoxygenase (6-*trans* leukotriene B_4_ and 5-HETE), and 15-lipoxygenase (15-HETE). Notably, levels of each of these pro-inflammatory metabolites of arachidonic acid were reduced in VAT from Ntn1^Δmac^ mice fed HFD, although the decrease in PGD2 did not reach statistical significance (Figure 7). Interestingly, although we observed a decrease in specialized pro-resolving lipid mediators (e.g., resolvin D2, resolvin E2 and maresin 2) in the VAT of HFD-fed WT mice compared to lean control mice, netrin-1 deletion in macrophages did not reverse this effect of HFD-feeding. Other lipid mediators, such as 17-HDHA, 14-HDHA, 12-HEPE and RvD5 were reduced in WT HFD as compared to chow controls but showed no genotype-specific differences (data not shown). Together, these data suggest that netrin-1 deletion in macrophages reduces the levels of pro-inflammatory lipid mediators in VAT known to contribute to chronic inflammation and insulin resistance in obesity.

## DISCUSSION

During the expansion of adipose tissue, inflammatory processes may be important for regulating adaptive responses, however the unresolved inflammation associated with obesity is thought to lead to metabolic dysfunction and insulin resistance [57,58]. Macrophages accumulate prominently in the obese VAT, and recent insights from single cell transcriptomics have revealed the diversity of macrophage phenotypes that emerge in response to this metabolic stress [23,24,52]. However, the signals that regulate the phenotypic differentiation and accumulation of these distinct macrophage pools are poorly understand. Our study shows that the neuroimmune guidance cue netrin-1 dynamically shapes the macrophage subsets that accumulate in the obese adipose tissue to sustain adipose tissue inflammation and metabolic dysfunction. Using conventional techniques, such as immunohistochemistry and flow cytometry, we show that macrophage-specific deficiency of netrin-1 caused a 35%–50% contraction of F4/80^+^ and CD64^+^ macrophages in the adipose tissue of HFD-fed mice. Further in depth analyses of VAT macrophage subpopulations using single cell RNA-seq showed that deficiency of netrin-1 specifically reduced resident and *Lcn*-hi macrophage subsets, while the major macrophage population in obesity was expanded. Furthermore, pseudotime resolution of macrophage transcriptional dynamics in obese adipose tissue revealed an altered macrophage trajectory in the absence of netrin-1, with the majority of monocyte-derived macrophages assuming a phenotype characterized by increased expression of genes specialized in lipid handling, fatty acid uptake and intracellular transport. Interestingly, compared to HFD-fed WT mice, Ntn1^Δmac^ mice showed reduced adiposity and increased expression in the VAT of genes involved in adipocyte lipolysis, fatty acid uptake and β-oxidation, and adaptive thermogenesis (*Prdm16*, UCP-1), suggestive of a functional increase in adipocyte-macrophage lipid flux that supports adipose tissue homeostasis. Consistent with these beneficial changes in adipose tissue inflammation and lipid homeostasis, Ntn1^Δmac^ mice showed improved insulin sensitivity compared to WT mice, suggesting that macrophage-specific targeting of netrin-1 may hold promise for the treatment of chronic inflammation and metabolic dysfunction in obesity.

In the obese adipose tissue, macrophages serve as a source of cytokines (e.g., TNFα, IL-1β), chemokines (CCL2), and inflammatory lipids (e.g., LTB_4_, PGE_2_) that recruit additional immune cells to amplify the inflammatory response and promote insulin resistance [1). Endogenous danger signals in the obese adipose tissue, such as high FFA concentrations and oxidative stress, trigger macrophage innate immune signaling pathways including toll-like receptors [59] and the NLRP3 inflammasome that regulates IL-1β secretion. In addition, these factors increase macrophage expression of netrin-1 and Unc5b [37,38], which can act in an autocrine manner to promote macrophage survival and chemostasis [37–39]. We postulated that the local secretion of netrin-1 by ATMs acts a as “stay put” signal that contributes to a maladaptive immune response in obesity by sustaining the cycle of non-resolving inflammation. Indeed, we show that deletion of netrin-1, specifically in myeloid cells, reduced VAT macrophage burden and decreased systemic and local markers of inflammation, including circulating levels of IL-6 and VAT levels of pro-inflammatory metabolites of arachidonic acid (e.g., prostaglandins and leukotrienes) known to promote persistent inflammation and altered lipid metabolism in obesity [60,61].

In lean adipose tissue, most tissue macrophages originate from yolk-sac macrophages prior to birth independently of bone marrow derived monocytes [62]. Consistent with this, our monocyte-macrophage trafficking studies show very little monocyte recruitment into the adipose tissue of lean mice. However, the recruitment of labeled monocytes into the adipose tissue is markedly increased upon HFD-feeding, and labeled monocyte-derived macrophages persist in the obese adipose tissue 14 days later. Notably, we observed no difference in monocyte recruitment to VAT in obese WT and Ntn1^Δmac^ mice, but fewer labeled macrophages persisted in the obese VAT of Ntn1^Δmac^ mice 14 days later, consistent with roles for macrophage-secreted netrin-1 in sustaining macrophage survival and retention in obese VAT. In addition to monocyte-derived macrophage recruitment and retention, the proliferation of resident adipose tissue macrophages also increases in obesity [7]. Interestingly, our single cell RNA-seq data indicate that loss of macrophage-derived netrin-1 results in a marked decrease in the resident adipose tissue macrophage subpopulation, suggesting that netrin-1 also contributes to the expansion of this macrophage subpopulation in obesity. Taken together, these data suggest that macrophage-derived netrin-1 contributes to the maintenance of both the monocyte-derived and resident adipose tissue macrophage pools in obese adipose tissue.

In the adipose tissue, lipid metabolism, inflammation and tissue homeostasis are inextricably intertwined. Recent studies have unveiled important roles for macrophages in maintaining lipid homeostasis in the expanding adipose tissue. ATMs are thought to buffer the increased concentration of lipids in the expanding adipose tissue by participating in lipid uptake, trafficking, storage and catabolism. Adipocytes can release lipid laden-exosomes that deliver triacylglycerides locally to macrophages and drive their differentiation in obesity [22]. In addition, adipocytes secrete FFAs liberated through the sequential actions of the neutral lipases ATGL and HSL, and these FFAs can be taken up by tissue macrophages, catabolized by β-oxidation, or released to the circulation. Our pseudotime analysis of single cell transcriptomes indicated that the majority of ATMs from HFD-fed WT mice showed a transcriptional response indicative of a high demand for protein synthesis and metabolic processes, consistent with the anabolic pressure produced by obesity. Interestingly, in HFD-fed Ntn1^Δmac^ mice there was a reprogramming of the ATM phenotype that resulted in higher expression of genes involved in lipid homeostasis. In the absence of netrin-1, monocyte-derived macrophages assumed an altered trajectory with activation of gene programs specialized in lipid handling, fatty acid uptake and intracellular transport, and lipid droplet activity. Interestingly, these changes in macrophage phenotype in HFD-fed Ntn1^Δmac^ mice were accompanied by a modest decrease in adiposity compared to WT mice. Investigation of the underlying mechanisms showed increased VAT expression of genes involved in both lipolysis (*Atgl, Hsl*) and FA oxidation (*Pparγ, Pgc1α*) as well as markers of AT beiging (*Prdm16*, UCP-1) in obese Ntn1^Δmac^ mice. In addition, we observed reduced plasma levels of FFA in Ntn1^Δmac^ mice compared to their WT counterparts, suggesting that increased lipolysis may be balanced by energy expenditure through fatty acid oxidation and uncoupled respiration. These findings, combined with the results of our single cell RNA-seq and pseudotime analyses, suggest that macrophage-specific netrin-1 deficiency improves lipid homeostasis in the VAT of obese mice. In sum, our results suggest that reductions in both adipose tissue fat mass and inflammation may contribute to the improvement in insulin sensitivity observed in mice with macrophage-specific deletion of netrin-1.

Our study used LysM-Cre transgenic mice to drive myeloid-specific deletion of netrin-1. Recent studies using LysM-Cre-TdTomato mice have shown that in addition to macrophages and neutrophils, LysM is expressed in a subset of neurons [63], raising the possibility that *Ntn1* may be targeted in some neurons in *Ntn1*^fl/fl^ LysMCre^+/–^ mice. TdTomato expression was detected in approximately 10% of neurons in the motorcortex and 20% in the cerebellum, while expression was absent in the Gyrus dentarus and CA1/2 regions of the hippocampus [63]. During development, netrin-1 protein is made by ventricular zone neuroepithelial progenitors and transported to the lateral margins of the spinal cord where it functions as a growth substrate for axons [64]. Genetic deletion of ventricular zone-derived netrin-1 results in defects in neural circuit organization and profound pathological consequences similar to those observed in full body deletion of *Ntn1*, which is lethal. Notably, we find that *Ntn1*^fl/fl^ *LysMCre*^+/–^ offspring are born at the expected Mendelian ratio, and we observe no developmental or behavioral abnormalities (including feeding and leptin levels]. However, our studies cannot exclude the possibility that LysMCre may delete *Ntn1* from some neurons in these mice. Future studies using models of inducible deletion of myeloid *Ntn1* in adult mice, such as CX3CR1-ERT2-cre*Ntn1*-fl/fl mice, will be useful to specifically target netrin-1 in macrophages after development.

Collectively, our findings extend previous studies showing that deficiency of netrin-1 in bone marrow derived cells reduced the accumulation of macrophages in the adipose tissue and improved insulin sensitivity during obesity [37], and confirm that macrophages are the major source of netrin-1 driving this phenotype. Although macrophage-specific targeting of netrin-1 is a promising approach to break the cycle of chronic inflammation in the obese adipose tissue, it remains to be tested whether broad netrin-1 targeting approaches, such as the use of anti-netrin-1 antibodies would be beneficial in this setting. In addition to its roles in chronic inflammation, we and others have shown that netrin-1 expression by endothelial or epithelial cells can protect from inflammation due to infection and ischemia [30–36,65]. In those contexts, netrin-1 contributes to the barrier function of the endothelium or epithelium by dampening leukocyte entry into tissues during transient ischemia of the gut, kidney or heart [30–36,65]. Thus, the dual functions of netrin-1 in protective and maladaptive inflammation [66] need to be closely considered in the development of netrin-1-directed therapeutics to avoid undesirable consequences. As chronic inflammation is now recognized to be a contributing factor in many other common diseases, including cardiovascular diseases, arthritis, and neurodegeneration, further study of factors such as netrin-1 that prolong inflammation will be needed to develop novel immune-directed therapies for deleterious inflammation.

## Supplementary Material

supplemental File 1
