# Supplementary material for: Netrin-1 Alters Adipose Tissue Macrophage Fate and Function in Obesity
Source: Immunometabolism. Author manuscript; Available in PMC 2019 Aug 19. (PMC6699780; doi:10.20900/immunometab20190010)
Supplement: supplemental File 1 [file NIHMS1045320-supplement-supplemental_File_1.pdf]

Supplementary Figure 1

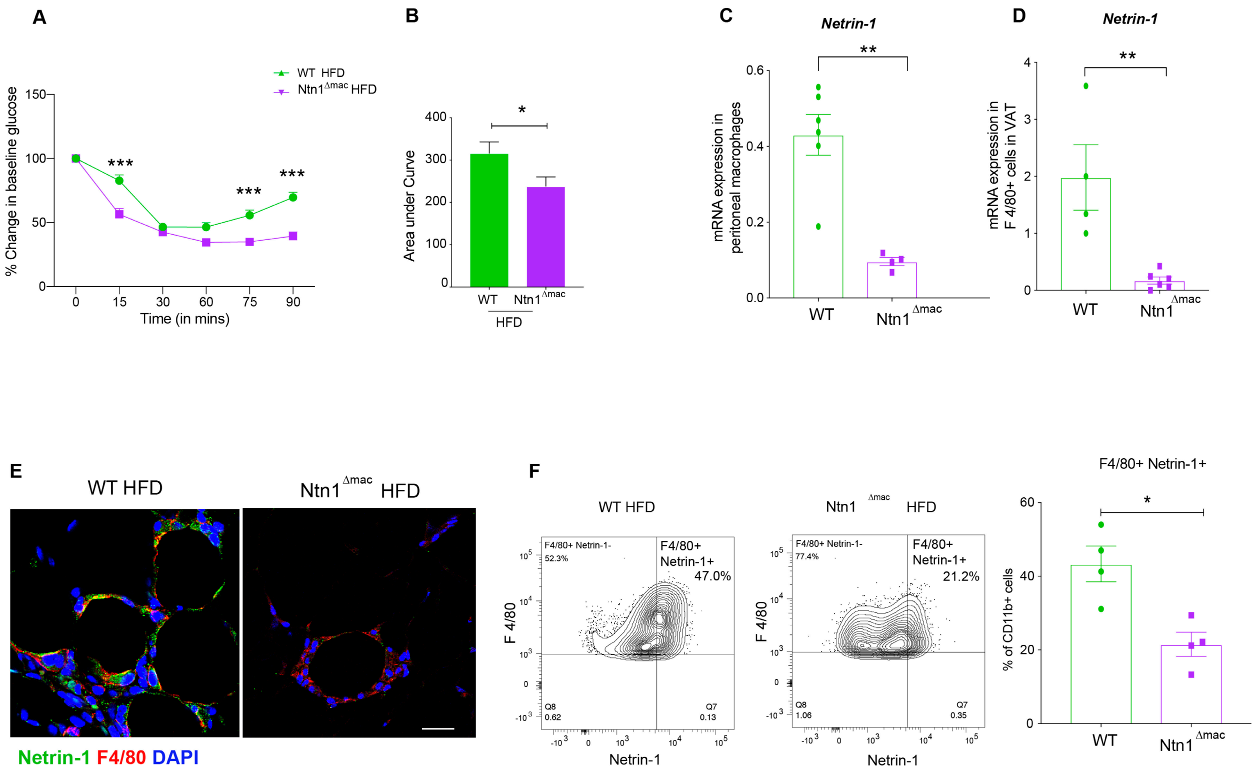

Figure S1. Insulin tolerance test plotted as (**A**) percentage of basal blood glucose as a function of injection time, (**B**) area under curve for percentage of basal blood glucose in Ntn1$^{\Delta mac}$ or WT mice fed chow or HFD for 20 weeks. qPCR analysis of Netrin-1 mRNA in (**C**) peritoneal macrophages form WT or Ntn1$^{\Delta mac}$ mice and (**D**) F4/80^{+} macrophages sorted from VAT from HFD fed- WT or Ntn1$^{\Delta mac}$ mice. n = 4–5 mice per group. **E)** Representative images of F4/80^{+} netrin-1+ stained macrophages of VAT sections of WT and Ntn1$^{\Delta mac}$ mice fed HFD. Scale bar = 100 uM. (**F**) Flow cytometric quantification of F4/80+ Netrin-1+ from digested VAT from HFD fed-WT or Ntn1$^{\Delta mac}$ mice. n = 4–5 mice per group. Data are the mean ± SEM; *p < 0.05, **p < 0.01, ***p < 0.001 (unpaired t-test).

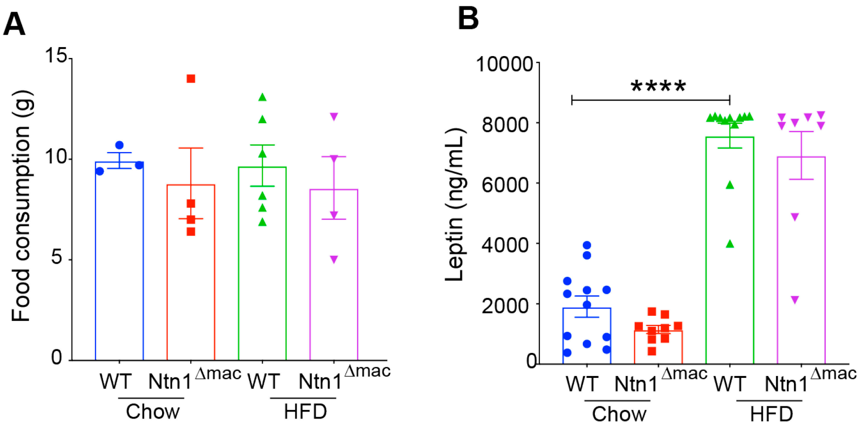

Figure S2. (**A**) Food consumption of individually housed mice over 72 h. $n = 5$/group, (**B**) Plasma leptin levels in Ntn1$^{\Delta mac}$ or WT mice fed chow or HFD for 20 weeks. Data are the mean ± SEM; *$p < 0.05$, **$p < 0.01$, ***$p < 0.001$ ****$p < 0.0001$ (one-way ANOVA with post-hoc Sidak's test).

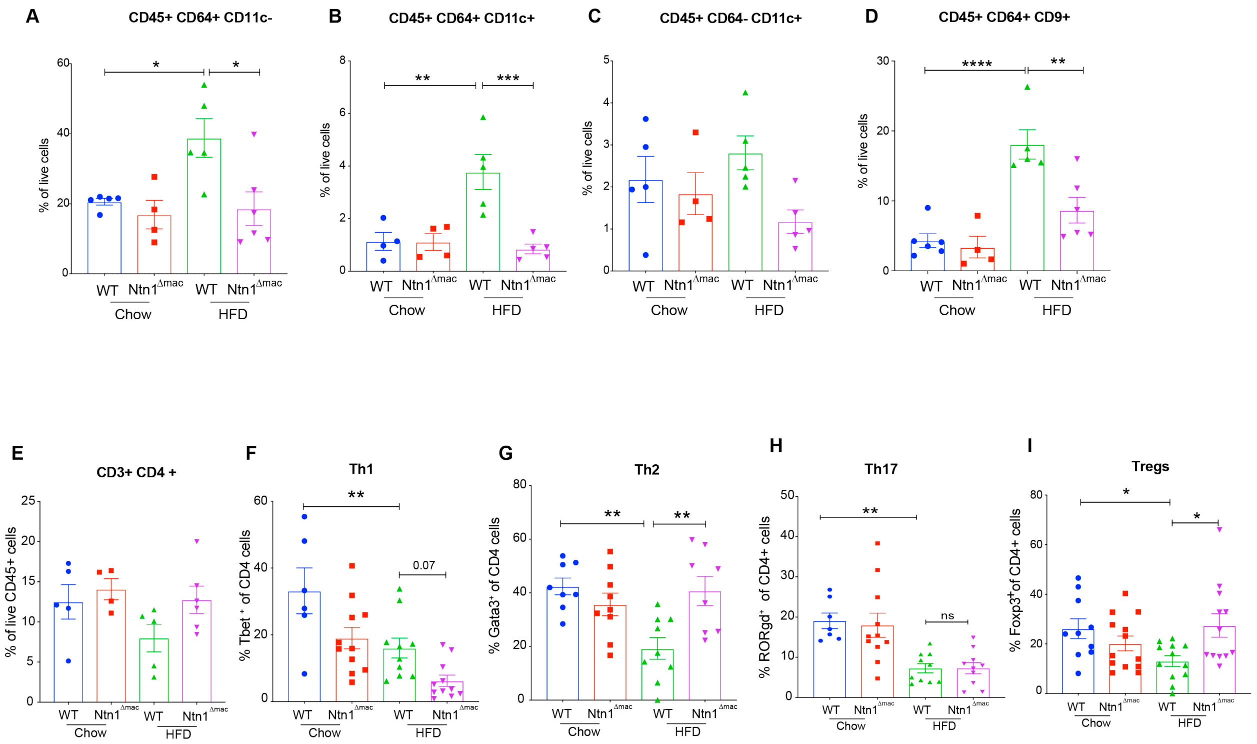

Figure S3. Flow cytometric quantification of (**A**) CD45^{+} CD64^{+}CD11c^{-} cells, (**B**) CD45^{+}CD64^{+}CD11c^{+} cells, (**C**) CD45^{+}CD64^{-}CD11c^{+} cells, **(D)** CD45^{+}CD64^{+}CD9^{+} cells, **E)** CD3^{+}CD4^{+} cells, (**F**) Tbet+ (Th1) cells, **G)** Gata3+ (Th2) cells, (**H**) RORgd^{+} (Th17) cells, (**I**) Foxp3^{+} (Tregs) cells in digested VAT in Ntn1$^{\Delta mac}$ or WT mice fed chow or HFD for 20 weeks. Data are the mean ± SEM; *$p < 0.05$, **$p < 0.01$, ***$p < 0.001$ ****$p < 0.0001$ (one-way ANOVA with post-hoc Sidak's test).

Supplementary Figure 4

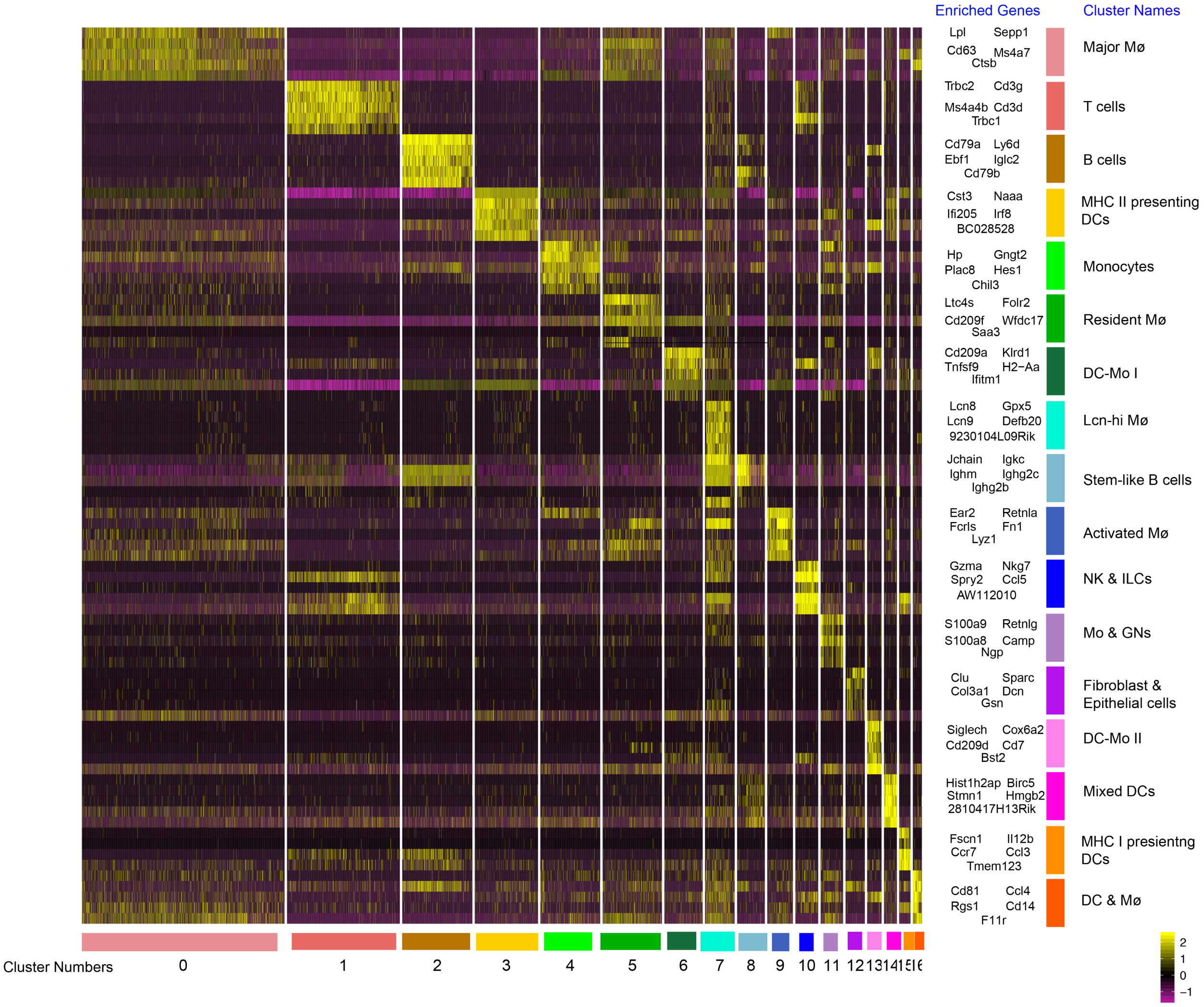

Figure S4. Heatmap showing the 5 most highly expressed genes per cluster (n = 17) identified from single-cell RNA-sequencing of $CD45^{+}$ cells from VAT from WT and $Ntn1^{\Delta mac}$ mice fed chow and HFD. Data were analyzed by SEURAT.

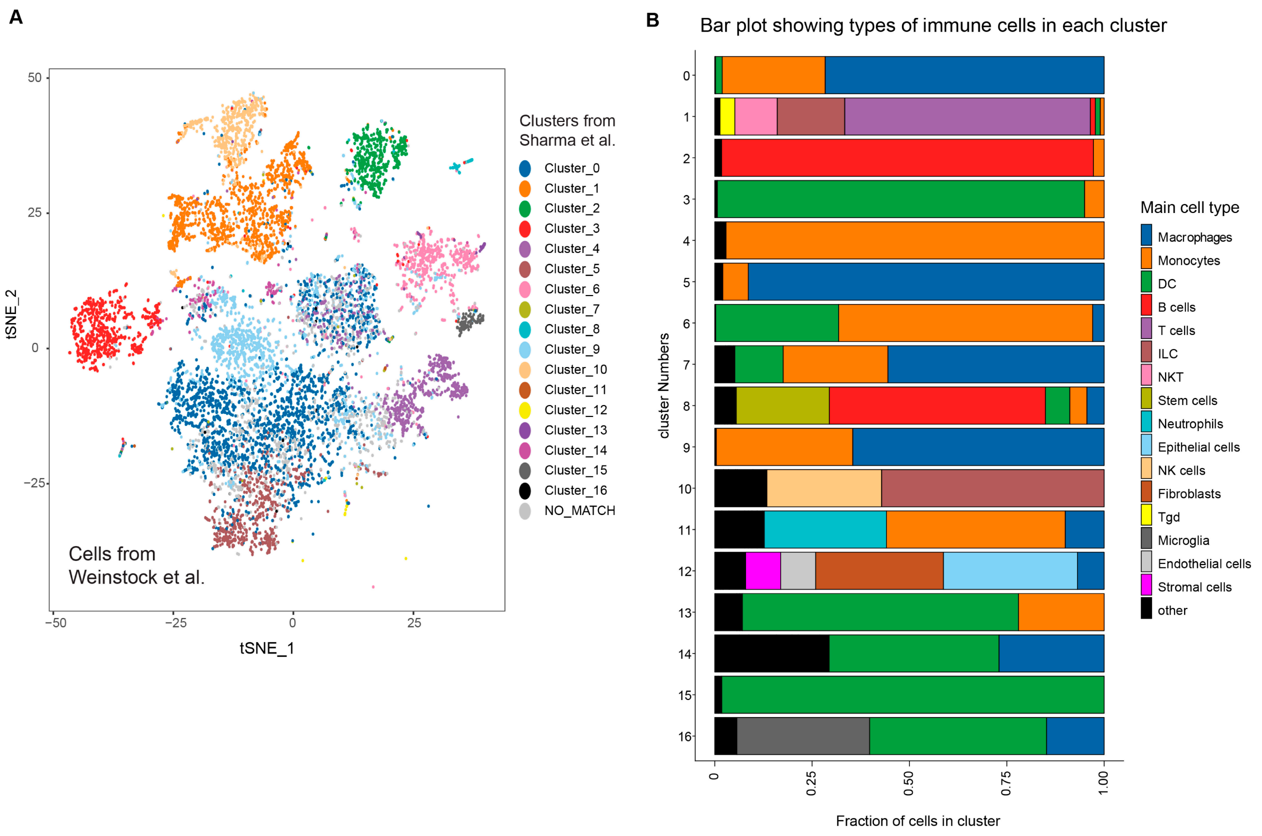

Figure S5. (**A**) Overlay of t-SNE plot of single-cell RNA-sequencing of CD45^{+} cells from VAT of HFD-fed mice from Weinstock et al. colored by the closest match in our single-cell RNA-seq dataset. Average expression profiles of the 17 clusters from WT and Ntn1$^{\Delta mac}$ mice fed chow and HFD were used as a reference dataset to annotate cells from Weinstock et al. using the R package SingleR. Any cells with an annotation p-value greater than 0.1 were categorized as "NO_MATCH"; 1,113 cells in the Weinstock dataset did not have a significant match, accounting for 11.2% of the total cells. (**B**) Cell type distribution in each cluster, assigned by SingleR, using the transcriptome of CD45+ cells from VAT of WT and Ntn1$^{\Delta mac}$ mice fed chow and HFD.

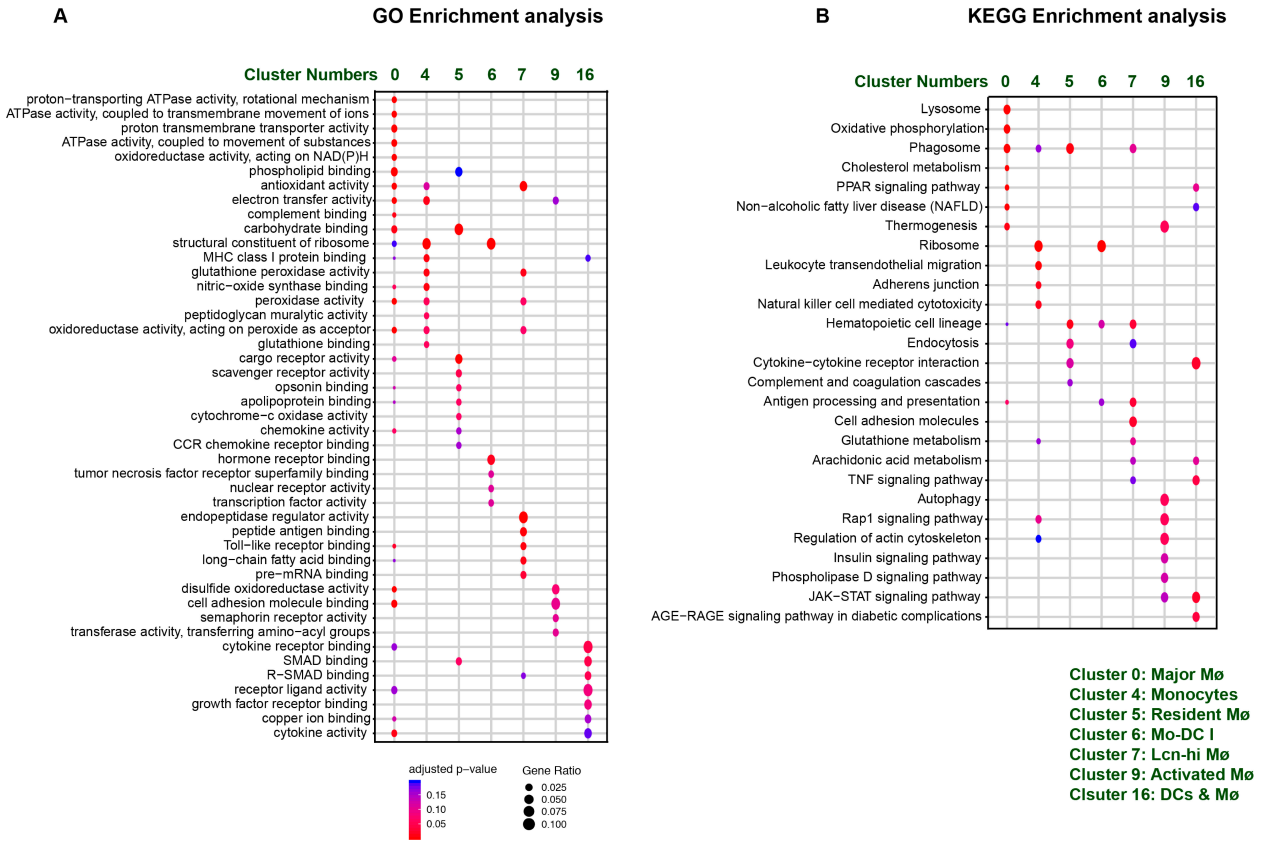

Figure S6. (**A**) GO enrichment pathway analysis, and (**B**) KEGG function analysis of monocyte and macrophage clusters identified from single-cell RNA-sequencing of $CD45^{+}$ cells from VAT of WT and $Ntn1^{\Delta mac}$ mice fed chow and HFD.
